# A Potential Effect of Circadian Rhythm in the Delivery/Therapeutic Performance of Paclitaxel–Dendrimer Nanosystems

**DOI:** 10.3390/jfb14070362

**Published:** 2023-07-11

**Authors:** Tânia Albuquerque, Ana Raquel Neves, Milan Paul, Swati Biswas, Elena Vuelta, Ignacio García-Tuñón, Manuel Sánchez-Martin, Telma Quintela, Diana Costa

**Affiliations:** 1 CICS-UBI—Health Sciences Research Centre, Universidade da Beira Interior, Avenida Infante D. Henrique, 6200-506 Covilhã, Portugal; tania.albuquerque@ubi.pt (T.A.); ana.neves@ubi.pt (A.R.N.); tquintela@fcsaude.ubi.pt (T.Q.); 2 Department of Pharmacy, Nanomedicine Research Laboratory, Birla Institute of Technology & Science-Pilani, Hyderabad Campus, Jawahar Nagar, Medchal, Hyderabad 500078, Telangana, India; milan.paul@hyderabad.bits-pilani.ac.in (M.P.); swati.biswas@hyderabad.bits-pilani.ac.in (S.B.); 3 Servicio de Transgénesis, Nucleus, Universidad de Salamanca, 37008 Salamanca, Spain; elena.vuelta.r@gmail.com (E.V.); adolsan@usal.es (M.S.-M.); 4 IBSAL, Instituto de Investigación Biomédica de Salamanca, 37007 Salamanca, Spain; ignacio.garcia@gmail.com; 5 Departamento de Medicina, Universidad de Salamanca, 37008 Salamanca, Spain; 6 Unidad de Diagnóstico Molecular y Celular del Cáncer, Instituto Biología Molecular y Celular del Cáncer (USAL/CSIC), 37007 Salamanca, Spain; 7 UDI-IPG-Unidade de Investigação para o Desenvolvimento do Interior, Instituto Politécnico da Guarda, 6300-559 Guarda, Portugal

**Keywords:** apoptosis, Bmal1 silencing, cancer therapy, caspases, circadian rhythm, nano-delivery systems, PAMAM

## Abstract

The circadian clock controls behavior and physiology. Presently, there is clear evidence of a connection between this timing system and cancer development/progression. Moreover, circadian rhythm consideration in the therapeutic action of anticancer drugs can enhance the effectiveness of cancer therapy. Nanosized drug delivery systems (DDS) have been demonstrated to be suitable engineered platforms for drug targeted/sustained release. The investigation of the chronobiology-nanotechnology relationship, i.e., timing DDS performance according to a patient’s circadian rhythm, may greatly improve cancer clinical outcomes. In the present work, we synthesized nanosystems based on an octa-arginine (R8)-modified poly(amidoamine) dendrimer conjugated with the anticancer drug paclitaxel (PTX), G4-PTX-R8, and its physicochemical properties were revealed to be appropriate for in vitro delivery. The influence of the circadian rhythm on its cellular internalization efficiency and potential therapeutic effect on human cervical cancer cells (HeLa) was studied. Cell-internalized PTX and caspase activity, as a measure of induced apoptosis, were monitored for six time points. Higher levels of PTX and caspase-3/9 were detected at T8, suggesting that the internalization of G4-PTX-R8 into HeLa cells and apoptosis are time-specific/-regulated phenomena. For a deeper understanding, the clock protein Bmal1—the main regulator of rhythmic activity, was silenced by Clustered Regularly Interspaced Short Palindromic Repeats (CRISPR) technology. Bmal1 silencing was revealed to have an impact on both PTX release and caspase activity, evidencing a potential role for circadian rhythm on drug delivery/therapeutic effect mediated by G4-PTX-R8.

## 1. Introduction

Although the understanding of cancer has increased over the past decades, it continues to be a leading cause of death worldwide and one of the major health scourges of the 21st century [1]. For decades, the main goal of oncology research has been the development of efficient therapies to eliminate cancer cells by the induction of programmed cell death. The apoptosis process can occur through two different pathways (intrinsic and extrinsic) both leading to the activation of caspases, proteins responsible to initiate and control this mechanism [2].

More recently, nanotechnology has been intensely explored for cancer treatment. Unlike conventional chemotherapeutics, nanoscale delivery systems have shown many advantages, such as improved stability and biocompatibility, precise targeting of tumor cells, increased cellular uptake, reduction in side effects, and drug resistance [3,4]. Therefore, research in the nanotechnology field offers safer and more efficient possibilities for the constant improvement of cancer therapy [5]. Several nanocarriers with distinct physiochemical properties and compositions have been designed for antitumor drug release. Some of those include polymer-based vectors, liposomes, micelles derived from polymers, carbon nanotubes, solid lipid nanoparticles, and magnetic nanoparticles [6]. To be significantly effective, they should be able to overpass biological barriers and target tumor tissues without losing substantial activity upon blood circulation [7]. Later, after cellular uptake, they should ideally be capable of endosomal escape and specific organelle targeting of the therapeutic agents [8].

Dendrimers are synthetic polymeric nanomaterials applied in both gene and drug delivery [9]. They are recognized as versatile carriers due to their unique structural features: a spherical polymeric core surrounded by multiple well-ordered geometrical branching molecules, where drugs can be encapsulated, and a periphery that can be functionalized with different ligands to enhance their stability, targeting ability, and tissue penetration [10]. These three-dimensional tree-like structures can be classified into “generations” (G) concerning the number of the branching in the dendrimer [11]. With an increase in generations, the molecular weight and number of reactive surface groups increase as well, creating dendrimers with different properties and possibilities of applications. Poly (amido amine) dendrimers (PAMAM), in particular, have been one of the most commonly used and have shown promising results for the delivery of poorly soluble anticancer drugs such as PTX [12], methotrexate (MTX) [13], doxorubicin [14], and docetaxel [15]. To further improve the cellular internalization of such constructs, Cell-Penetrating Peptides (CPPs) can be added. CPPs are small peptides originating from different sources that possess remarkable properties of cell penetration, facilitating the translocation of therapeutic agents into cells [16,17]. Cationic peptides are a class of CPPs that easily interact with the phospholipids, negative in charge, in the cell membrane, facilitating membrane translocation. For instance, octa-arginine (R8), a short-chain peptide with eight arginine residues was found to mediate intracellular uptake of the nano-constructs leading to the intracellular release of a high amount of PTX promoting its anticancer efficacy [12,18]. Chronotherapy has also gained much attention in the cancer research field as an emergent strategy to enhance current cancer treatments. It is based on the principle that the safety and efficacy of chemotherapeutics can be dependent on the time of their administration [19]. So far, several clinical trials have applied chronotherapeutic protocols for cancer treatment and have confirmed an optimal timing for therapy, improving its efficacy, diminishing drug toxicity, and enhancing patient survival [20,21]. In fact, it is well documented that the circadian rhythm exerts a role in cancer progression since its dysfunction was associated with the activation of intracellular inflammatory and oncogenic signaling pathways [22,23]. On the other side, circadian rhythms modulate many cellular and physiological processes including drug metabolism, absorption, transport, distribution, detoxification, and subsequent toxicity and efficacy of the treatment. The study of circadian fluctuations of the molecules that are involved in these processes allows for the estimation of a more suitable time for treatment administration. The coordination of circadian rhythms is controlled by biological clocks found in nearly every cell in the body. A molecular clock machinery consists of clock genes/proteins that integrate regulatory feedback loops in which they regulate their own expression. The main components of the clock include brain and muscle ARNT-like1 (BMAL1), circadian locomotor output cycles protein kaput (CLOCK), the proteins period (PER1, PER2, and PER3), and cryptochrome (CRY1 and CRY2). The transcription factor CLOCK:BMAL1 forms a dimer to promote the expression of PER and CRY, which, in turn, act as repressors of CLOCK:BMAL1 in the nucleus. The outcome is the circadian oscillation of many genes and proteins involved in several physiological processes, within a period of about 24 h [24]. A correlation between clock gene expression and the apoptotic effect of anticancer agents has been reported [25]. Slat et al. found that the anticancer drug temozolomide (TMZ) attained a maximum effect close to the daily peak of Bmal1 expression. Moreover, the deletion of the core clock gene abolished the rhythmicity in TMZ-induced apoptosis in vitro [25]. Another study using in vitro and in silico models linked irinotecan cytotoxicity, a drug for colorectal cancer, to clock gene Bmal1 expression, putting in evidence a role for circadian rhythms in cancer treatment [26].

An attractive approach to overcome the limitations of current chemotherapeutics would be the combination of chronobiology with nanotechnology; however, few works have addressed the influence of the circadian clock in the efficiency of nanoformulations, namely, the cell entry of the vector, its release pattern and therapeutic effect. In a previous work, the effect of the circadian clock on the cellular uptake of nanoparticles based on polyethylenimine (PEI) to deliver MTX and p53-plasmid DNA to HeLa and C33A cell lines was investigated [27]. The results revealed a specific time point, after cell synchronization, for the high performance of the drug/gene co-delivery system. In this work, we intended to continue the research on this topic and bring advances to the chronobiology-nanotechnology relationship. Pursuing this challenge, we synthesized a PAMAM dendrimer of generation 4 (G4), in which PTX and R8 were coupled (G4-PTX-R8). After the physicochemical characterization of the dendrimer nanosystem, it was tested whether the time point of delivery affected the amount of both cell-associated PTX and caspase activity in HeLa cells. Furthermore, we successfully performed Bmal1 silencing on HeLa cells to clarify the impact of the circadian clock on cellular uptake and apoptosis. Our data demonstrated significant differences, in the profile of these phenomena, between wild-type and knockout cells, suggesting a potential role for the circadian clock in the performance of a G4-PTX-R8 delivery system. 

## 2. Materials and Methods

### 2.1. Materials

Dulbecco´s Modified Eagle´s Medium with Ham’s F-12 Nutrient Mixture (DMEM-F12) with L-glutamine was purchased from CORNING (New York, NY, USA). Cervical cancer HeLa cells were obtained from ATCC (Manassas, VA, USA). 3-(4,5- dimethylthiazol-2-yl)-2,5-diphenyltetrazolium bromide (MTT) was supplied by Sigma-Aldrich (St. Louis, MO, USA). PTX was purchased by MedChemExpress (Monmouth Junction, NJ, USA). For G4-PTX-R8 synthesis, the dendrimer of fourth generation containing ethylenediamine core groups and surfaced amino groups (G4 PAMAM) was acquired from Dendritech (Midland, MI, USA). N-hydroxysuccinimide and N-(3-Dimethylaminopropyl)-N-ethyl carbodiimide hydrochloride (EDC. HCl, 98%) were supplied by Sigma Aldrich Chemicals (St. Louis, MO, USA). Finally, the cellulose dialysis membrane (MWCO 3.5 KDa) was obtained from Spectrum Laboratories, Inc. (Dominguez, CA, USA). All other commercially purchased solvents and chemicals were of analytical grade.

The reagents required for CRISPR-Cas9 technology (single guide RNAs (crRNAs), ATTO-labelled tracrRNA, and Cas9) were obtained from IDT (IA, Coralville, IA, USA); Buffer R and the primers for Bmal1 were purchased from Invitrogen (Carlsbad, CA, USA). The ApoAlertTM Caspase-3 Colorimetric assay kit was purchased from Clontech Lab (Orange, CA, USA), and Caspase-Glow^®^ 9 Assay from Promega (Madison, WI, USA). 

### 2.2. Preparation of PTX-2-Hemisuccinate (PTX-SA)

**Figure 1:**
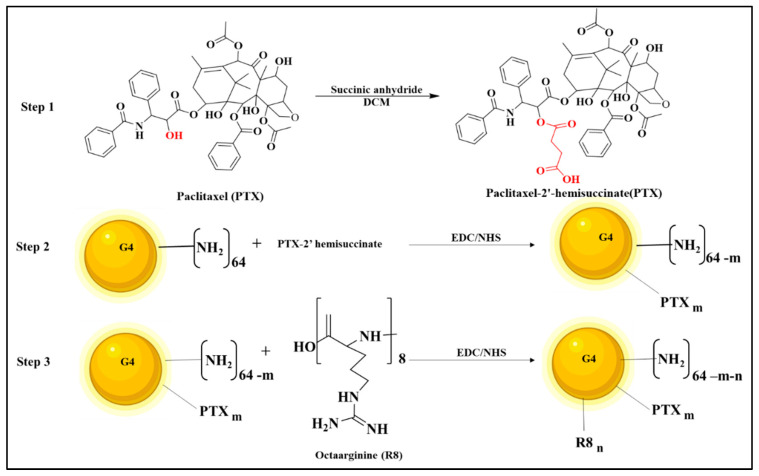
Illustration representing the main three steps in the preparation of the multifunctional G4-PTX-R8 complex.

The steps for the formation of the G4 dendrimer conjugate are illustrated in Figure 1. The PTX-2´-hemisuccinate was synthesized by activating the 2′-OH group of PTX using succinic anhydride (SA). Briefly, to 25 mg of PTX in Dichloromethane (DCM, 2 mL), 4.4 mg of SA was added, in the presence of dry pyridine, at a mol ratio of 1:1.5. This reaction was continuously mixed for 3 days [28]. Next, the PTX-SA was extracted from the aqueous reaction mixture using ethyl acetate, and the solvent was evaporated under vacuum, resulting in a white powder with an ~85% yield. The product was identified by thin-layer chromatography using silica-coated plate and solvent system. Dichloromethane: methanol. 85:15.

### 2.3. Preparation of PAMAM G4-PTX 

The cross-linking agent 1-ethyl-3-(3-dimethylaminopropyl)-carbodiimide/N-hydroxysuccinimide (EDC/NHS) was used to activate the PTX-2-hemisuccinate in 2 mL of Dimethylformamide (DMF). The reaction occurred for 6 h at room temperature [29]. 

Following that, 50 mg of G4 PAMAM Dendrimer in DMF was transferred to the activated PTX-NHS ester, dropwise under nitrogen. 

The PAMAM G4: PTX-SA mol ratio was 1:4. The reaction continued overnight. Next, using a vacuum, the DMF was vaporized. Then, the solution was added to a dialysis membrane (3500 Da MWCO) and dialyzed for 48 h.

Afterward, the solution was lyophilized, and a white powder was obtained. A yield of ~75% was obtained. 

### 2.4. Preparation of PAMAM G4-PTX-R8

Into the solution of R8 (9.87 mg, 0.0078 mmol) and trimethylamine (20 µL) in DMF, EDC/NHS (3 mol equivalent of the R8) G4-PTX (40 mg, 0.0026 mmol) was combined following the activation step. The reaction was continued overnight, followed by evaporation of DMF. Finally, using a cellulose ester membrane (MWCO. 3.5 KDa), the crude product was dialyzed against water, and the final mixture was lyophilized. A pure product was obtained with a yield of ~82%. An illustration with the main steps of the preparation of the dendrimer complex is represented in Figure 1.

### 2.5. Nuclear Magnetic Resonance (NMR) Spectroscopy of PAMAM G4-PTX-R8 Complexes

*NMR* was employed to characterize the developed conjugates, using an NMR Spectrometer (400 MHz, Bruker, Billerica, MA, USA). The samples of G4, PTX-SA, and G4-PTX-R8 (10 mg/mL) were dissolved in the solvent CDCl3 and the spectra were obtained after 128 scans [30]. 

### 2.6. Circular Dichroism (CD) Spectroscopy

The secondary structure of R8 and G4-PTX-R8 was analyzed, under a nitrogen atmosphere using a CD spectrometer (Shimadzu, Japan) with a scanning range of 200–260 nm. The spectrum was compared with the standard R8 to confirm the presence of R8 in the formulation [31]. 

### 2.7. Zeta Potential and Particle Size

The average zeta potential and particle size of the G4-PTX-R8 were determined by the principle of dynamic light scattering using Zetasizer 3600 (Malvern Instruments Ltd., Malvern, UK). The zeta potential, particle size, and polydispersity index (PdI) were measured before and after lyophilizing the sample. The sample was diluted in Milli-Q water before analysis [32].

### 2.8. Morphological Analysis Using SEM

For the study of the G4-PTX-R8 surface morphology, Scanning Electron Microscopy (SEM, NOVA NANOSEM 450) analysis was performed. 

For sample preparation, a thin layer was evenly distributed and fixed to an aluminum stub with an adhesive carbon tape. Then, the stub was coated with the desired gold thickness and analyzed with a 20 kV accelerating voltage [33].

### 2.9. Cell Culture

For the in vitro experiments, cervical cancer HeLa cells were grown in 25 cm^3^ T-flasks as described before [27]. HeLa cells (including knockout HeLa cells) were grown with DMEM-F12 supplemented with 10% heat-inactivated fetal bovine serum (FBS) and 1% (V/V) of a penicillin/streptomycin solution (100 µg/mL). Cells were incubated at 37 °C, under a 5% CO_2_ humidified atmosphere, to promote cellular growth until reaching confluency.

### 2.10. Cytotoxicity Study

G4-PTX-R8 dendrimer powder was dissolved in DMSO:water 0.8:0.2 for posterior studies.

To evaluate the cytotoxicity of free PTX and the G4-PTX-R8 dendrimer on HeLa, an MTT assay was performed. HeLa cells were seeded at a density of 5 × 10^3^ cells/well in 96-well plates, and the day before the experiments, the culture medium was changed to DMEM-F12 supplemented with 10% FBS without antibiotics to enhance the cellular uptake [34,35].

Different formulations of PTX and G4-PTX-R8 ranging from 0 to 100 ug/mL were prepared and added to the cells during 6 h. Afterward, medium was replaced, and cells were allowed to grow at 37 °C and 5% CO_2_ for 48 h. After that period, MTT was added to each well at a final concentration of 0.5 mg/mL and incubated for a further 4 h. Furthermore, the culture medium was discarded and dimethyl sulfoxide (DMSO, 100 µL) was added to dissolve the formazan crystals. Thereafter, the plates were shaken and a purple color was produced.

The absorbance was then read at 570 nm using a Benchmark Microplate Reader (BioRad, Vienna, Austria). As a negative control of the experiment, wells with no treated cells were considered and cells treated only with DMSO were used as positive control. The medium without cells was set up as zero absorbance and used for spectrophotometer calibration. 

The cell viability (%) was therefore calculated in relation to the control wells by the formula: [A]test/[A]control × 100, [A]test being the absorbance of the test sample and [A]control the absorbance of the positive control sample. 

Moreover, the half maximal inhibitory concentration (IC_50_) was assessed for free PTX and the G4-PTX-R8 complex.

### 2.11. In Vitro Studies to Acess Circadian Rhythms Impact on Cellular Uptake of the Nanosystem

For in vitro studies, HeLa cells were seeded at a density of 5 × 10^4^ cells/3.8 cm^2^ in 12-well microplates and grown until reaching 70–90% of confluence. Before the experiments, cells were synchronized for 2 h with 0.1 μM of dexamethasone. Then, the medium was removed and cells returned to normal conditions [27]. Cells were incubated with free PTX or the G4-PTX-R8 complex at the IC_50_ concentration, calculated for G4-PTX-R8.

The delivery was performed at T0 (immediately after cell synchronization) and every 4 h during 24 h (T4; T8; T12; T16; and T20). After 6 h of incubation, cells were either collected at the different time points for the cell-associated PTX absorbance measurement or cells returned to usual culture condition and left to grow during 48 h for subsequent assays.

### 2.12. Cell-Associated PTX 

To evaluate the cellular uptake of PTX in HeLa cells at the different time points, cell-associated PTX was measured. After collecting the cells, the pellet was rinsed in phosphate-buffered saline (PBS) and collected. Then, cell lysis was performed by incubation with a 1% Triton X-100 solution for 30 min at 37 °C. Lastly, the solution was pipetted into a black plate, and intracellular PTX was determined by reading the absorbance at 230 nm using a Shimadzu UV-Vis 1700 spectrophotometer (Biorad). Cells without treatment were used as control [34].

### 2.13. Bmal1 Silencing on Hela Cell Line

The gene editing technique CRISPR-Cas 9 was used with the purpose of studying the effect of Bmal1 deletion on the efficacy of the G4-PTX-R8 system at all the 6 time points. Two crRNAs (Integrated DNA Technology) were designed to generate a small deletion (140 bp) targeting exon 8 (Supplementary Material, Table S1). For the complex formation, equimolar amounts were mixed to a final duplex concentration of 44 μM. Then, the mixture was heated to 95 °C for 5 min and then the temperature was ramped down to 25 °C on a thermocycler. Next, 18 pmol of Cas9 (Integrated DNA Technology) was added to the initial duplex reaction (22 pmol) to a final volume of 1 μL for electroporation and the mixture was set at room temperature (RT) for 1 h. Before electroporation, 2 μL of 10.8 μM of Electroporation Enhancer and 9 μL of cell suspension were added to the final reaction. An amount of 2 × 10^5^ cells was electroporated in a Neon TM Transfection System (Invitrogen, Waltham, CA, USA) following the manufacturer’s instructions and using the electroporation parameters of 1005 V, 35 ms, and 2 pulses.

The day after transfection, cells were observed under confocal microscopy to confirm the efficiency of electroporation. Subsequently, cells were sorted with a FACSaria (BD Biosciences, San Jose, CA, USA). This step was performed to select the gene-targeted cells by separation of the Bmal1 cell population from the control with only Cas9. Results were analyzed using FlowJo software. 

After the sorting, cells were collected, and one single cell was seeded in a new plate and left to grow into colonies. The confirmation of the knockout harboring clones was first assessed by conventional PCR (primer sequence on Supplementary Material, Table S2). The AllPrep DNA Kit (Qiagen) was used for the extraction of Genomic DNA according to the manufacturer’s protocol. The PCR products were cleaned with the NZYGelpure kit (NZYTech, Lisbon, Portugal) and sequenced by the Sanger method [36].

### 2.14. Caspase-3 and Caspase-9 Activity Assay

Caspase-3 and caspase-9 activity were measured on wild-type and knockout HeLa cells following the provided instructions. In brief, after the incubation with free PTX or G4-PTX-R8, cells were allowed to grow for 24 h. An incubation with 1 μM of staurosporine, under the same conditions, was used as a positive control.

The caspase-3 activity was determined by using a caspase colorimetric assay Kit (ApoAlertTM), by measuring the absorbance at 405 nm of p-nitroaniline (p-NA) after cleavage from the substrate DEVD-pNA. A Luminescent Assay (Caspase-Glow^®^ 9) was applied for the determination of Caspase-9 activity following the provided protocol [34]. 

### 2.15. Statistical Analysis 

For the comparison between the distinct experimental groups, one-way/two-way analysis of variance (ANOVA) and Bonferroni test were employed. The data were analyzed in GraphPad Prism v.8.01 software (San Diego, CA, USA). Statistically significant differences were considered for a p-value below 0.05 (* *p* ≤ 0.05; ** *p* ≤ 0.01; *** *p* ≤ 0.001; **** *p* ≤ 0.0001).

The rhythmicity in PTX intracellular uptake and caspase activity was analyzed with CircWave v1.4 analysis software (Dr. Roelof A. Hut, Groningen, The Netherlands) by a harmonic regression method, assuming a period of 24 h, and with alpha set at 0.05. Statistically significant rhythms were considered for a *p*-value below 0.05.

## 3. Results

### 3.1. Preparation and Characterization of Multifunctional Dendrimer Conjugate

The multifunctional dendrimer conjugate was synthesized as described in Figure 1 and characterized by NMR spectroscopy, Fourier transform infrared spectroscopy (FTIR), X-ray Photoelectron spectroscopy (XPS), CD Spectroscopy, Gel Permeation Chromatography (GPC), Zeta potential and Particle size determination, and SEM (detailed description available in Supplementary Material).

**Figure 2:**
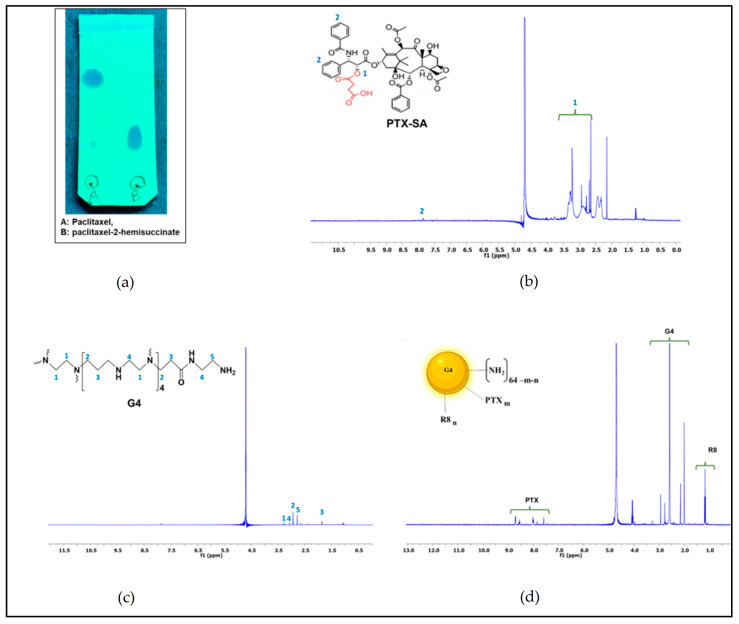
Thin-layer chromatography of PTX-SA conjugate (**a**), 1H-NMR spectra of PTX-SA (**b**), PAMAM G4 (**c**), and G4-PTX-R8 (**d**).

Figure 2b shows the 1H-NMR spectra of PTX-SA displaying a peak at 2.5–2.8 (δ), which indicates the coupling of succinic acid to the PTX. The spectra of G4 and G4-PTX-R8 present a signal at ppm 7–8.5 (δ), which corresponds to the aromatic groups of the PTX. In Figure 2c,d, the signals from the dendrimer protons are observed at ppm 1.5–3.5 (δ). Finally, the signals visible at ppm 1–1.5 (δ) relate to the methylene protons of R8 [12]. Furthermore, FTIR and XPS validated the conjugation of PTX and R8 to the dendrimer (Figures S1 and S2 and Table S3, all presented in Supplementary Material)).

**Figure 3:**
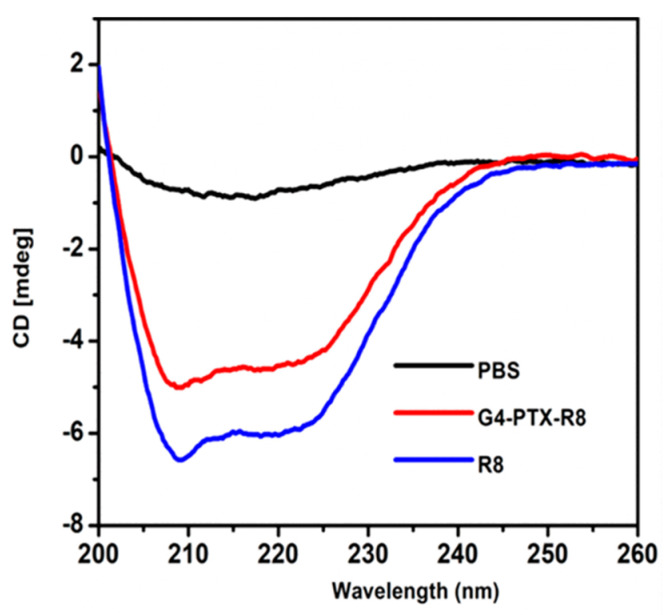
Representation of CD spectra of R8 and G4-PTX-R8. The presence of R8 in the final formulation was confirmed, as well as the structural integrity following conjugation.

To further confirm the presence of R8 in the formulation, CD Spectroscopy was performed. The alpha-helix and beta-sheet values obtained for standard R8 were 70.71 ± 0.79 and 10.21%, respectively. The R8-conjugated polymer, G4-PTX-R8, displayed the values as 59.32 ± 0.12 and 14.32%, respectively (Figure 3). The decrease in the CD signal in the G4-PTX-R8 conjugate indicated the binding of molecules to the peptide, which induced its conformational change. However, the secondary structure remains the same with the predominance of alpha-helical structure. 

The relative molecular weights of the synthesized product were determined by using GPC. The average molecular weight of the dendrimer complex was 19313 Da (Table S4, Supplementary Material). The GPC data analysis also indicated that 2.48 molecules of R8 were conjugated to one molecule of G4-PTX (Figure S3, Supplementary Material) in the stated experimental conditions.

**Figure 4:**
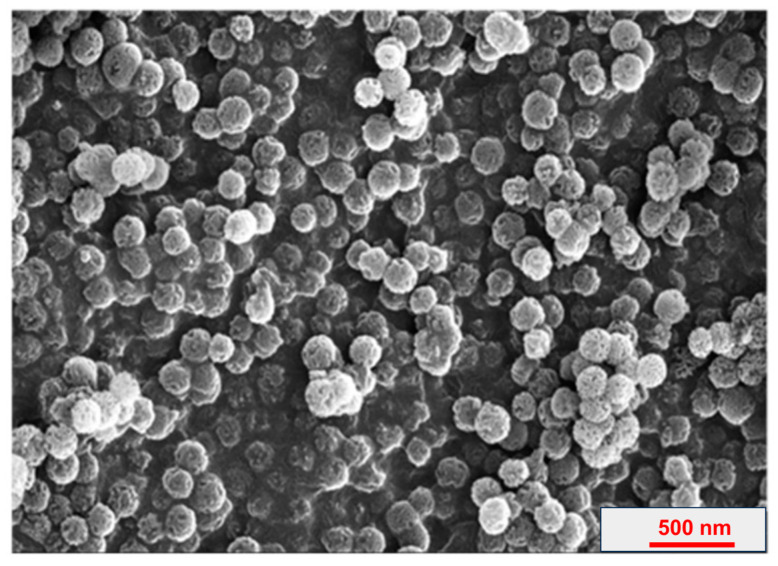
SEM image of PAMAM G4-PTX-R8 dendrimer.

Finally, the zeta potential and particle size of G4-PTX-R8 formulations are presented in Table 1. The morphological analysis using SEM (Figure 4) displayed uniform spherical morphology of the dendrimer, and the zeta potential revealed the positive surface charge of the conjugate. 

### 3.2. Cytotoxicity Studies

**Figure 5:**
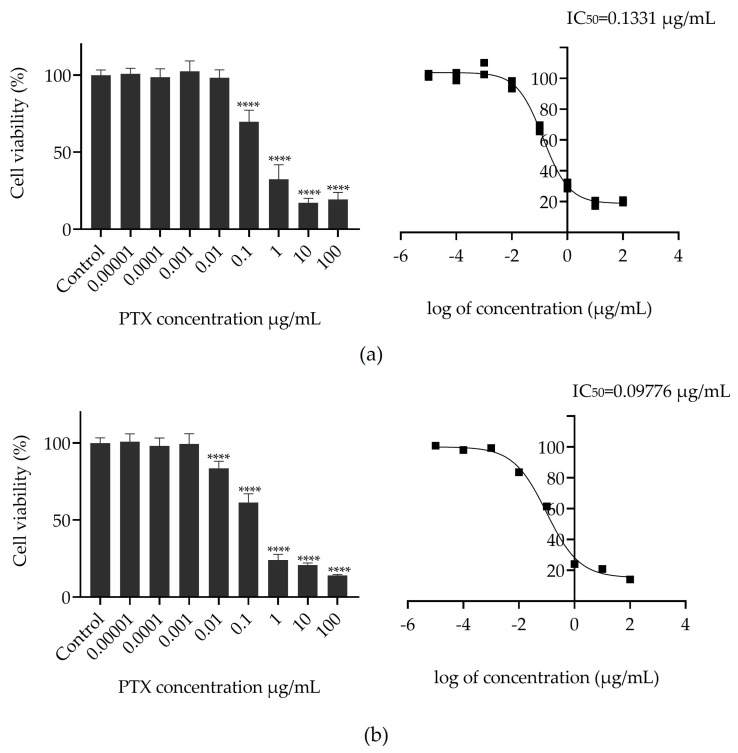
Dose–response curves and cellular viability of HeLa cervical cancer cells after 48 h of treatment with free PTX (**a**) and the G4-PTX-R8 complex (**b**). Each data point represents the mean value calculated with the data obtained from independent measurements (n = 3, mean ± SD), which were analyzed by one-way ANOVA with the Bonferroni test (**** *p* < 0.0001 relative to control).

To determine the efficacy of the PTX conjugate in inhibiting cancer cell viability, an MTT assay was performed in HeLa cells. Cells were treated with free PTX or G4-PTX-R8 at concentrations ranging from 0 to 100 µg/mL and incubated for 48 h, as presented in Figure 5. Cells that did not receive the treatment were taken as a positive control for cellular viability. The toxicity was accessed by calculating the IC_50_ value from the different concentrations using a nonlinear curve fitting algorithm. The results of the assay demonstrated that there is evidence of a cellular viability decrease in time as the PTX concentration increases (Figure 5). 

The determination of the median inhibitory concentration is used to estimate how effective a given anticancer drug is at reducing cellular viability. The value of IC_50_ is lower for G4-PTX-R8, meaning that a lower dose is required to produce the same toxicity as free PTX. The subsequent experiments were conducted based on the IC_50_ value achieved for G4-PTX-R8.

### 3.3. The Impact of Circadian Rhythm on Cellular Uptake/Internalization

#### 3.3.1. Determination of Cell-Associated PTX 

**Figure 6:**
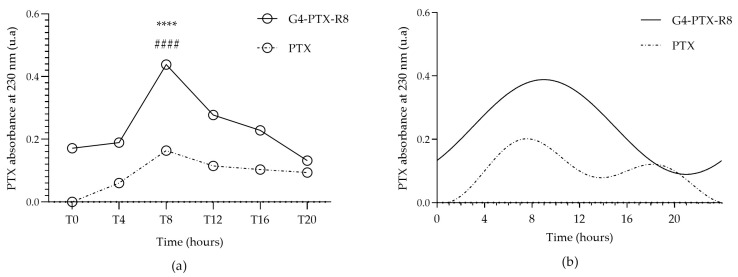
Quantification of cell-associated PTX absorbance. The absorbance of PTX was measured 6 h after the cells were incubated with either free PTX or the G4-PTX-R8 dendrimer complex, at the considered time points (**a**). Each point represents the mean value calculated with the data obtained from independent measurements (n = 3, mean ± SD), which were then analyzed by two-way ANOVA followed by Bonferroni test. A higher absorbance of PTX was noticed for G4-PTX-R8 at T8 and this difference was considered statistically significant in relation to other time points (**** *p* < 0.0001). Furthermore, the difference in PTX values was also statistically significant between free PTX and the G4-PTX-R8 dendrimer complex for T8 (#### *p* < 0.0001). Circadian oscillations were considered significant for both free PTX and the G4-PTX-R8 complex after the analysis with CircWave (*p* < 0.001) (**b**).

Cell synchronization was confirmed after analyzing the expression of Bmal1 over 24 h (Figure S4). To evaluate the impact of the circadian rhythm on PTX cellular uptake/internalization, the released PTX in the cytosol of HeLa cells after incubation with the G4-PTX-R8 dendrimer or free PTX was measured for each time point indicated above. Figure 6a shows the obtained results. From the presented results, regarding the cell-associated PTX absorbance measure, we can infer that both free drug and dendrimer-associated drug are internalized by HeLa, however, in a different extension and differently over time. 

Comparing the various time points considered, the highest levels of PTX were noticed for T8 and T12 in HeLa cells with statistically significant differences in relation to other time points. A complete statistical analysis is found in Supplementary Material (Table S5). 

After that, and to conclude if the differences are circadian significant, the oscillation patterns of cell-associated PTX absorbance were obtained by CircWave analysis and are presented in Figure 6b. For both free PTX and the G4-PTX-R8 dendrimer the oscillations observed were considered significant (*p* < 0.05; Table S6 in Supplementary Material).

#### 3.3.2. Generation of a Bmal1 Knockout Cell Line

To investigate whether the rhythmicity in PTX internalization is dependent on the circadian clock, we knocked out the Bmal1 gene on HeLa cells using CRISPR-Cas9; Bmal1 is essential for rhythmic gene expression in the circadian system.

For the experimental protocol, we selected 2 crRNA and a tracrRNA labeled with the fluorescent dye ATTO, which allows for the visual analysis of transfected cells and posterior cell isolation. After one single cell seeding and having sufficient colonies, a portion of cells was collected, and we proceeded with the experiments to confirm the silencing. 

First, genomic DNA was extracted, RT-PCR was performed, and the PCR products were visualized under UV light. The deleted fragment from exon 8 was about 140 bp (Figure S5 in Supplementary Material) and Sanger DNA sequencing confirmed successful gene edit.

Furthermore, Western blot confirmed a silencing of about 60% (Figure S6, in Supplementary Material).

#### 3.3.3. Bmal1 Silencing Effect on Cellular Internalization of PTX

**Figure 7:**
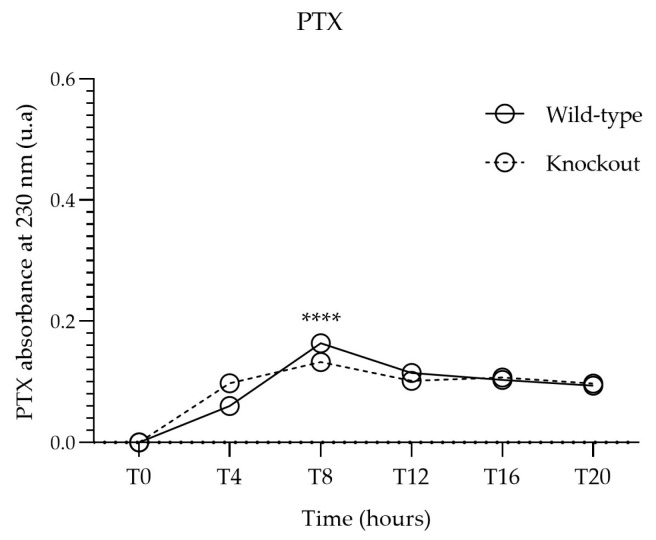
Comparison of cell-associated PTX absorbance between wild-type and Bmal1 knockout HeLa cells incubated with free PTX. Each point represents the mean value calculated with the data obtained from independent measurements (n = 3, mean ± SD), which were then analyzed by two-way ANOVA followed by Bonferroni test. Significant differences were observed for T4, T8, and T12 (Table S7, **** *p* < 0.0001).

After confirming Bmal1 silencing in HeLa cells, we followed the experiments with the wild-type and knockout-generated cell lines and proceeded as described before to quantify the internalized PTX. In Figure 7, the PTX absorbance profile of PTX alone in wild-type and knockout cells is represented. It can be noticed that the knockout of Bmal1 did not remarkably affect PTX internalization, although slightly significant differences were detected between the two conditions for T4, T8, and T12 (Table S7 in Supplementary Material). Moreover, the CircWave curve of PTX absorbance in knockout HeLa indicates rhythmicity between time points (Figure S7a). Nonetheless, a compromised circadian clock on HeLa cells does not seem to greatly interfere with the uptake of the drug, and differences cannot be estimated for the low amount of PTX alone that these cells can incorporate.

**Figure 8:**
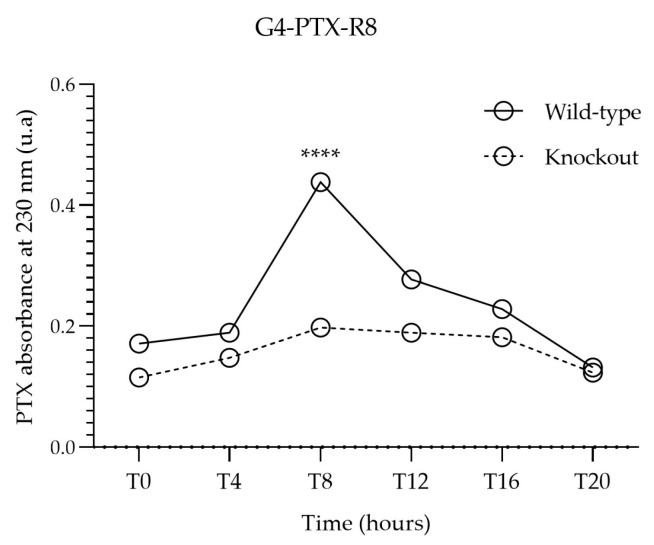
Comparison of cell-associated PTX absorbance between wild-type and Bmal1 knockout HeLa cells incubated with G4-PTX-R8. Each point represents the mean value calculated with the data obtained from independent measurements (n = 3, mean ± SD), which were then analyzed by two-way ANOVA followed by Bonferroni test. Significant differences were mainly observed for T8 and T12 (Table S7, **** *p* < 0.0001).

On the contrary, a pronounced difference was observed when cells were exposed to G4-PTX-R8 (Figure 8). In Bmal1-knocked-out cells, the presence of PTX dropped significantly at T8. Likewise, variations in drug internalization between time points in knockout cells are less visible in comparison to the wild-type ones, even though a pattern can be traced (CircWave analysis, Figure S7b, Supplementary Material). 

#### 3.3.4. The Role of Bmal1 on Therapeutic Effect Mediated by G4-PTX-R8

**Figure 9:**
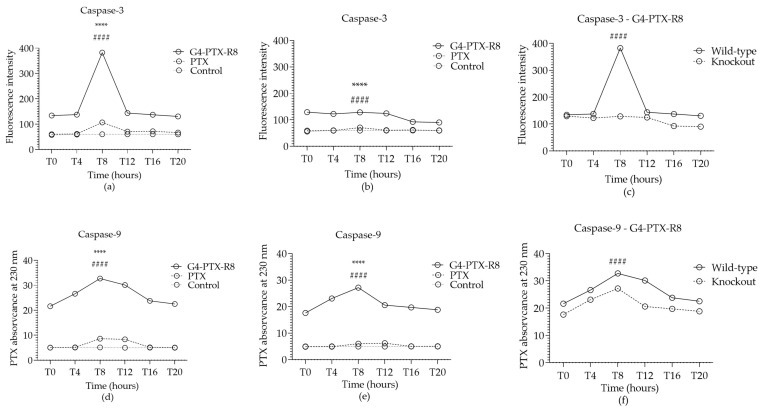
Caspase-3 (**a**–**c**) activity and caspase-9 (**d**–**f**) activity in wild-type (**a**,**d**) and knockout HeLa cells (**b**,**e**) after 24 h of treatment with the free PTX or G4-PTX-R8. Cells were incubated with 1 μM of staurosporine to be used as a positive control, while untreated cells were considered as a negative control. Each point represents the mean value calculated with the data obtained from independent measurements (n = 3, mean ± SD), which were then analyzed by two-way ANOVA followed by Bonferroni test. Statistically significant differences were found between time points (**** *p* < 0.0001) and between PTX and G4-PTX-R8 dendrimer complex or between wild-type and knockout cells (#### *p* < 0.0001). The complete statistical analyses can be found in Table S8. Circadian oscillations were considered significant after the analysis with CircWave (Figure S8).

The impact of the circadian rhythm on the potential therapeutic effect displayed by the developed nanosystems was also studied. In line with this, in this work, it was explored if an induction on apoptosis mediated by PTX was achieved in a circadian manner, and, further, the influence of Bmal1 on caspase expression was analyzed. For that, the caspase-3 and caspase-9 activity was assessed in both wild-type and knockout HeLa cells. The results are presented in Figure 9. 

When comparing caspase activity mediated by the G4-PTX-R8 complex with the free PTX, for wild-type HeLa, higher levels of both caspase-3 and caspase-9 are observed at T8 but also at T12 for caspase-9 (Figure 9a,d), indicating a greater capability of G4-PTX-R8 to induce apoptosis at those specific time points. 

To further study the contribution of the circadian clock in apoptosis, caspase content was also analyzed on knockout cells (Figure 9b,e) and then compared to the data collected with wild-type cells for the delivery mediated by G4-PTX-R8 (Figure 9c,f). 

The Bmal1 silencing significantly reduced the activity of caspase-3 but not so extensively the activity of caspase-9, showing a less dependent circadian regulation. 

A higher absorbance of PTX was noticed for G4-PTX-R8 at T8 and this difference was considered statistically significant in relation to other time points (**** *p* < 0.0001). Furthermore, the difference in PTX values was also statistically significant between free PTX and the G4-PTX-R8 dendrimer complex for T8 (#### *p* < 0.0001).

## 4. Discussion

The PTX drug is a member of the taxane family that acts as an anticancer drug. Since its anticancer activity was discovered, it has been investigated for the treatment of a variety of carcinomas [37,38,39]. In its mechanism of action, it is a diterpenoid compound that stabilizes microtubules reducing the dynamics of tubulin necessary for cellular division and leading to the termination of the cell cycle with arrest at the G2/M phase. Despite being one of the most successful chemotherapeutics used in the clinic, it faces some problems with its use owing to its low aqueous solubility [40].

In this work, PTX was combined with a PAMAM dendrimer to improve its solubility and, consequently, enhance its therapeutic effect. The conjugation of R8 to a multifunctional dendrimer was also intended to enhance cellular entry. 

The positive charges provided by the amine groups of G4 and by the cationic peptide contributed to the positive zeta potential of the G4-PTX-R8 complex, favoring electrostatic interactions with the negatively charged cytoplasmic membrane and promoting its cellular internalization. 

Furthermore, the small size and PdI, as well as the uniform spherical morphology, are favorable properties for cellular uptake and were considered acceptable for drug delivery [41].

Furthermore, a potential role for the circadian rhythm in the delivery/therapeutic performance of the nanosystem was evaluated. The characterization studies proved the presence of both PTX and R8 in the conjugated form of the polymer, G4-PTX-R8. Moreover, they also confirmed the structural integrity following conjugation, which needs to be preserved for optimum receptor interaction of R8 in its intracellular target site.

In vitro experiments revealed that both the free drug and dendrimer-associated drug are internalized into HeLa cells. Due to its hydrophobicity, PTX might be integrated into the hydrophobic domain of the lipid membrane cell bilayer, reaching this way the cytoplasm. However, as expected, free PTX uptake into cells occurred to a less extent comparatively with the dendrimer-associated PTX. It is known that dendrimers as a delivery platform can improve drug therapeutic outcomes by facilitating their entry into cancer cells; therefore, PTX loading into a G4-R8 dendrimer may overcome its poor aqueous solubility and bioavailability [42].

The differences in cell-associated PTX absorbance over time suggest that there is a different time point where PTX reaches its highest cell internalization efficiency (T8 and T12) and that there is a time when these cells seem to be more likely to internalize the PTX drug. So far, few studies have investigated the effect of the circadian rhythm on the effectiveness of delivery systems for the release of therapeutic molecules. Kreuter was the first to investigate the role of chronobiology on the uptake and transport profile of nanoparticles into the brain of mice [43]. Concerning cancer therapy, PTX had been already considered for a chronotherapeutic protocol. PTX-loaded polymeric nanoparticles were tested in xenografted human lung cancer. Chonomodulated delivery of PTX-NPs showed dependence upon administration time, reaching an optimal level at 15 h after light onset [44]. In addition, the cytotoxic effect of PTX-NPs was lower compared to the PTX injection. Recently, our research group also demonstrated a possible circadian control over the internalization of a drug/gene co-delivery carrier, instigating further research on this matter [27]. 

In this work, we aimed to knock down the core clock gene on HeLa cells using CRISPR-Cas9 to clarify if the observed differences in PTX intracellular uptake among the distinct time points could, hypothetically, be influenced by the expression of Bmal1. Cas9 nuclease-based genome editing has become a standard technology that can be applied within cells and whole organisms. It can “switch-off” genes allowing for the generation of knockout cell lines via site-specific double-stranded breaks within a genome [45]. Here, we used the CRISPR-Cas9 genome editing system to knock out Bmal1 from HeLa cells. The silencing of the core clock gene has been of extreme importance to understanding how circadian rhythms can modulate several physiologic processes associated with diseases, thus allowing for new treatment options. In fact, mutations in clock genes or circadian rhythm desynchronization have been associated with a range of diseases [46]. As an example, recently, it was found that the intestinal exporter MRP2, involved in the disposition and removal of several drugs, exhibits diurnal oscillation in wild-type mice. However, the ablation of Bmal1 caused a reduction in Mrp2 mRNA and protein levels, and also affected the transport of the anticancer drug MTX [47]. Other authors have successfully knocked down Bmal1 [48,49]. 

In the present study, the knockout of Bmal1 affected PTX internalization when cells were treated with G4-PTX-R8, especially at T8. This result implies that the efficiency of the cellular uptake is to some extent dependent on a functional circadian clock. Nanosystems can be internalized into cells by distinct routes. For PAMAM dendrimers, the mechanisms of cellular uptake might vary with their generation, functionalization, and between different cell lines. Endocytosis (via clathrin and caveolae) and passive diffusion are reported to be the main mechanisms of cellular uptake. Still, their interaction with cell membranes remains poorly understood [50]. The possibility of circadian machinery playing a role in these mechanisms is yet to be investigated, but it may offer a totally new avenue of research toward novel therapeutic strategies.

To further explore if circadian rhythm could impact the therapeutic performance of the dendrimer, the induction of apoptosis was assessed by the quantification of caspase-3 and caspase-9 activity. Apoptosis exerts a prominent role in the treatment of cancer, and it is a process mediated by several signaling pathways. Failure in any step may promote tumor progression and resistance to anticancer therapies [2]. This programmed cell death process can be activated through intrinsic (mitochondrial) and extrinsic (death receptors) pathways depending on the nature of the signal. In any case, both pathways lead to the activation of cysteine proteases (caspases), causing the proteolysis of hundreds of proteins and changes to the plasma membrane that activates macrophage response [51]. Caspase-9 is an initiator caspase involved in an intrinsic pathway [52], whereas caspase-3 is the main executioner of apoptosis and is activated by both pathways [53]. Several anticancer drugs are known to target some of the stages in both apoptosis pathways, thus controlling growth/proliferation as an effective method to control cancer [54]. 

Concerning PTX’s ability to activate caspase activity, in the present work, high levels of both caspase-3 and caspase-9 were observed at specific time points for cells treated with G4-PTX-R8. Indeed, it is known that anticancer drugs can activate intrinsic and extrinsic pathways [54]. The prominent expression levels at T8 and T12 might be due to the higher levels of PTX internalized at these time points. For PTX alone, caspase-3 and caspase-9 activity are proximal to the control, exceptionally for T8 and T12 as well, reinforcing a possible circadian control in the cellular uptake of PTX and G4-PTX-R8. Nevertheless, treatment with free PTX does not seem to have significant anticancer action on HeLa cells.

The efficiency of an anticancer formulation might be dependent on the mechanism leading to cell death. The induction of apoptosis after PTX exposure is well documented in various cancer cell lines. As an example, in human leukemia NB4 cells, PTX induced a reduction in P53 and the activation of caspase-3 and caspase-9 for long treatment periods [55]. Likewise in human breast cancer cells, PTX-induced cell death mediated the activation of the intrinsic pathway of apoptosis [56]. Recently, the combination of PTX with other anticancer agents, enhanced apoptosis by the intrinsic pathway via ROS generation [57] in HeLa cells. Nonetheless, it is recognized that PTX can induce many ways of cell death and initiates apoptosis through multiple mechanisms/pathways [58]. 

Moreover, naked PAMAM dendrimers have also been studied as therapeutic options. They may lead to cancer cell apoptosis through the up-regulation of apoptotic biomarkers (caspase-3, caspase-8, and caspase-9) [59].

Regarding the circadian control of cell death, the time-of-day specificity of known anticancer drugs has been investigated recently in the human osteosarcoma U2OS cell line [60]. A screening of 126 drugs targeting various signaling pathways controlling cell survival, cell cycle control, and apoptosis was performed. Out of the 126 compounds, 62 exhibited daily rhythms of cytotoxicity and antiproliferative activity (accessed by IC_50_ calculation and revealing the adenosine 5′-triphosphate (ATP) amount in cells). Additionally, analyses of target genes for these drugs revealed that several of them also exhibit significant daily variations in expression, which indicates a circadian regulation of the cell cycle [60]. 

For knockout HeLa cells, Bmal1 silencing reduced the activity of caspase-3 but not so extensively the activity of caspase-9, revealing an impact on caspase activity. Indeed, it has been earlier demonstrated that some core clock genes are relevant for cell apoptosis. For instance, clock genes inhibited apoptosis on a human glioma cell line [61], whereas treatment with cisplatin caused a dual effect: the upregulation of clock expression, increasing proliferation; and the upregulation of Bmal1 expression, which increases apoptosis [62]. 

To study the time-specificity effect on the sensitivity of cancer cells to anticancer drugs, the deletion of core clock circadian clocks has been applied. So far, several reports have stated that Bmal1 is a relevant transcription factor capable of suppressing the proliferation, migration, and invasion of several cancer cells [63,64,65]. Tang et al. researched the role of the clock gene Bmal1 on tumor inhibition and PTX sensitivity in tongue squamous cell carcinoma. They concluded that after PTX treatment, Bmal1-overexpressing cells showed increased apoptosis and higher sensitivity to PTX in vivo [64]. The increased susceptibility to this anticancer drug was mediated by TERT (Telomerase Reverse Transcriptase), a downstream gene of Bmal1, whose activity was found to be under circadian control and widely activated in cancer [66]. The overexpression of Bmal1 in nasopharyngeal carcinoma cells (NPC) also allowed for similar conclusions. The upregulation of Bmal1 was found to inhibit cell proliferation, increase the sensitivity of NPC cells to radiotherapy, and increase the expression of p53 and p21, whereas the knockdown of Bmal11 had the opposite effect [66]. However, it seems that circadian proteins play different roles depending on the tissue and the applied treatment. In liver cancer cells, Bmal1 or Clock down-regulation induced apoptosis and arrested the cell cycle at the G2/M phase [67]. Similarly, Bmal1 knockdown suppressed the proliferation of malignant pleural mesothelioma [68]. Concerning caspase activity regulation, it has been shown, in glioblastoma cells, that the activity of caspase-3/7 was higher at the peak of Bmal1 expression and that Bmal1 loss resulted in ablation of the rhythm in TMZ-induced caspase activity [25]. Similarly, in U87MG glioblastoma cells some pro-apoptotic markers and caspase-3 were increased in Bmal1 overexpressing cells [64]. Therefore, our results are consistent with findings from the literature, evidencing a potential role for Bmal1 and the circadian clock in cancer cells’ sensitization to PTX and further enhanced apoptosis at specific time points. Here, we detected both caspase-3 and caspase-9 activities, which could be an indicator of the activation of the mitochondrial route of apoptosis. Curiously, Bmal1 depletion does not affect caspase-9 expression in the same magnitude as it does for caspase-3. It is also possible that the extrinsic pathway was involved in the response to PTX/G4-PTX-G8 treatment and, if so, in that case, caspase-3 could be activated by other caspases such as caspase-8. 

Given our findings, we believe that a way to make cancer therapy more effective should include the optimization of nanosystem design/development to ensure drug delivery at the appropriate time of the day, in accordance with the patient´s circadian rhythm. 

Needless to say, the suitable time for in vivo administration should take several factors into account. Upon administration, nanoparticles will be exposed to pharmacokinetic phenomena, many of which are under circadian regulation. All these factors are supposed to impact drug toxicity and efficacy and must be considered for chronotherapy applications to enhance cancer therapy outcomes. 

## 5. Conclusions

The attractive concept of “Chronotherapy” has been explored to enhance cancer therapy outcomes. In parallel, to overcome the major obstacles of conventional therapies, a variety of nanosystems have been considered for the targeted drug release to cancer cells.

In this work, we developed nanocomplexes based on a PAMAM dendrimer of generation 4 for PTX delivery into HeLa cancer cells. Regardless, these molecules can also face some challenges, such as membrane permeability. Consequently, the R8 peptide was successfully conjugated on the dendrimer surface. In our study, the efficiency of dendrimer cellular uptake was directly correlated with higher PTX absorbance found in the cytosol. We noted a superior PTX internalization when PTX was coupled with PAMAM and R8 rather than when used alone. Therefore, the dendrimer is a suitable vehicle for the release of poorly soluble drugs. 

To unveil the effect of the circadian rhythm on the efficiency of cellular internalization, we measured cell-associated PTX and caspase activity on wild-type HeLa and Bmal1-silenced cells. The obtained results demonstrated higher delivery of PTX at T8 and T12 and superior caspase-3 activity at T8, and at T8 and T12 for caspase-9 activity. As for caspase activity, only caspase-3 expression was compromised by the Bmal1 knockdown in HeLa cells. Also, the knockdown maintained a rhythmic pattern for PTX internalization and caspase activity, however, to a much less extent. We concluded that Bmal1 appears to be necessary for the efficient uptake of the dendrimer and for the execution of programmed cell death. Bmal1 can either promote the cellular internalization of PTX, leading to the activation of apoptosis, or can increase susceptibility to the drug, leading to increased apoptosis at a specific time point. Although our results do not cover a full understanding of the role of Bmal1, our work strongly suggests the influence of Bmal1 on the performance of PTX/G4-PTX-G8 cellular uptake, PTX delivery, and, consequently, apoptosis induction, and, thus, therapeutic effect. The reported data should instigate the design of novel delivery systems displaying a more precise targeting to cancer cells, at a more favorable time. If translated and implemented in the clinic, it could have an exponential impact on cancer therapy.

## Figures and Tables

**Table 1:** Size, PdI, and zeta potential value obtained for PAMAM G4-PTX-R8 dendrimer.

	Zeta Potential (mV)	PdI	Size (nm)
PAMAM G4-PTX-R8	+9.325	0.287	23.74 ± 0.454

## Data Availability

All data is provided in the principal document of the manuscript or as supplementary materials.

## Supplementary Materials

The following supporting information can be downloaded at: https://www.mdpi.com/article/10.3390/jfb14070362/s1, Figure S1: FTIR spectra of G4, PTX-SA, G4-PTX, and G4-PTX-R8; Table S1: crRNA sequences; Figure S2: XPS spectra of G4, G4-PTX, and G4-PTX-R8; Table S2: Primers designed for RT-PCR; Figure S3: GPC thermogram of G4, G4-PTX, and G4-PTX-R8; Table S3: XPS data of PAMAM G4-PTX-R8 dendrimer; Figure S4: CircWave analysis of bmal1 rhythmicity after HeLa cell synchronization; Table S4: GPC data of PAMAM G4-PTX-R8 dendrimer; Figure S5: Genomic map of Bmal1 showing the sequence of the deleted fragment from exon 8 in grey and RT-PCR products of the isolated clones; Table S5: Statistic analyses of Bonferroni’s multiple comparisons test for PTX internalization on HeLa cells; Figure S6: Western blot analysis of BMAL1 protein expression in HeLa cells; Table S6: Results of CircWave analysis of cell-associated PTX absorbance for each time point; Figure S7: Circadian oscillations are statistically significant as analyzed with CircWave for PTX and the G4-PTX-R8 complex; Table S7: Statistic analyses of Bonferroni’s multiple comparisons test for PTX and G4-PTX-R8 internalization on HeLa knockout cells; Figure S8: Circadian oscillations are statistically significant as analyzed with CircWave for caspase-3 and caspase-9 activity after incubation with PTX and G4-PTX-R8; Table S8: Statistic analyses of Bonferroni’s multiple comparisons test for caspase activity on wild-type and knockout after incubation with free PTX or G4-PTX-R8.
