# Supplementary material for: A Potential Effect of Circadian Rhythm in the Delivery/Therapeutic Performance of Paclitaxel–Dendrimer Nanosystems
Source: J Funct Biomater. 2023 Jul 11;14(7):362. doi: 10.3390/jfb14070362 (PMC10381694; doi:10.3390/jfb14070362)
Supplement: Supplementary file 1 [file jfb-14-00362-s001.zip › jfb-2476857-supplementary.pdf]

Supplementary Materials

Experimental Section

Synthesis and characterization of multifunctional dendrimer conjugate

Fourier transform infrared spectroscopy (FTIR)

The functional groups present in the conjugate were characterized using IR spectroscopy (Jasco-4200, USA). FTIR analysis was performed using KBr pellet method.26 The scanning range was kept from 4000 to 400 cm-1.

X-ray Photoelectron spectroscopy (XPS)

The chemical compositions were analysed by X-ray photoelectron spectroscopy (XPS, KAlpha, and Thermo-Fischer). The obtained XPS data were analysed using Advantage software.

Gel Permeation Chromatography (GPC)

The relative molecular weights of the synthesized product were determined by using Gel Permeation Chromatography (Waters Corporation, USA) using Ultrahydrogel linear (7.8 mm ID × 300 mm × 6 μ) size exclusion column. Double distilled water was used as a mobile phase at a flow rate of 1ml/min. The entire experiment was conducted in isocratic mode. The calibration curve was carried out using standard of shodex ranging molecular weight between 6300 to 530000 before analysing the sample. The sample peak was analysed by integrating and calculating using the calibration curve plotted earlier. Mn, Mw, Mp were determined, and the number of molecules attached to the dendrimer was reported.

Bmal1 silencing on HeLa cell line

Table S1: crRNA sequences.

crRNA BMAL 1	crRNA BMAL 2
CAACATGCAACGCAATGTCCAGG	CTAGAATGTTCCTATCCCTAAGG

Table S2: Primers designed for RT-PCR.

	Forward (5′-3′)	Reverse (5′-3′)
BMAL1-RT-PCR	CAGGGAAGCTCACAGTCAGA	CATTTGGCATCACGGCTGTT

Results

Synthesis and characterization of multifunctional dendrimer conjugate

Fourier transform infrared spectroscopy (FTIR)

FTIR spectroscopy was performed to confirm the conjugations of PTX, and R8 to the dendrimer. The presence of a peak at 2927 cm-1 indicates the presence of NH stretch. Next, the peak at 1758 and 1250 cm-1 corresponds to the amide carboxyl group stretch and C-N stretch, respectively (Figure 4). These peaks confirmed the conjugation of R8 and PTX on the dendrimer G4.

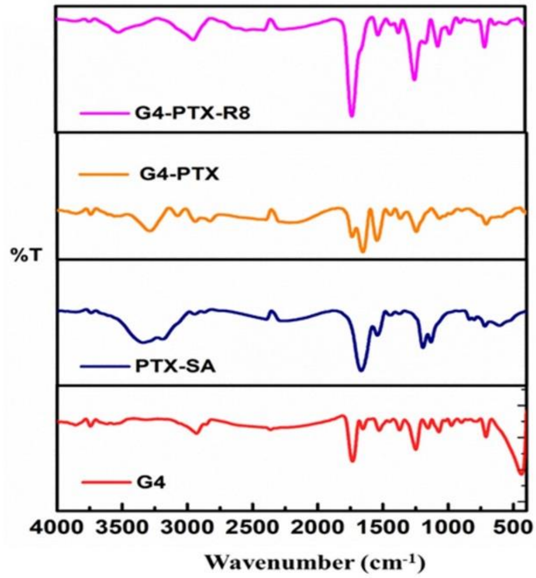

Figure S1: FTIR spectra of G4, PTX-SA, G4-PTX and G4-PTX-R8.

X-ray Photoelectron spectroscopy (XPS)

According to the XPS spectra, an 8.63 % increase in oxygen and 8.4 % decrease in the nitrogen in G4-PTX-R8 polymer was observed compared to G4 (Figure 7 and Table 3). The conjugation of oxygen-rich PTX-SA and R8 increased the oxygen content in the dendrimer surface. Similarly, the surface-amine content decreased in G4 following conjugation resulting in a gradual decrease in the nitrogen content in G4-PTX, and G4-PTX-R8. Overall, the result indicated successful conjugation of PTX and R8 moiety to the dendrimer.

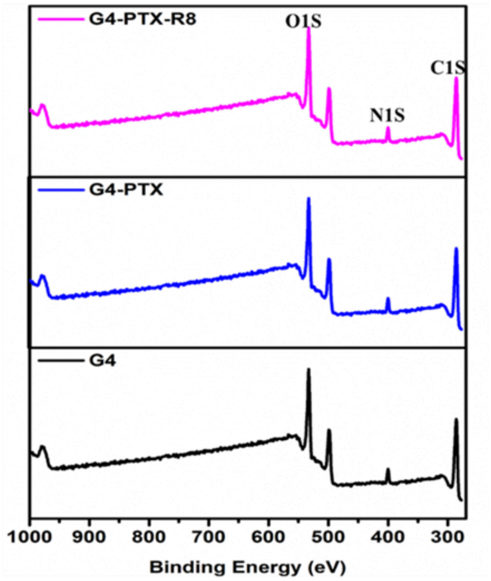

Figure S2: XPS spectra of G4, G4-PTX and G4-PTX-R8.

Table S3: XPS data of PAMAM G4-PTX-R8 dendrimer.

	**O1**	**N1**	**C1**
G4	18.33	14.89	66.78
G4-PTX	22.96	10.49	66.55
PAMAM G4-PTX-R8	26.96	6.49	66.55

Gel Permeation Chromatography (GPC)

Table S4: GPC data of PAMAM G4-PTX-R8 dendrimer.

	**Mw (Da)**	**Mn**	**Mp**	**PdI**	**Number of molecules attached**
PAMAM G4-PTX-R8	19313	15416	17969	1.235	2.48

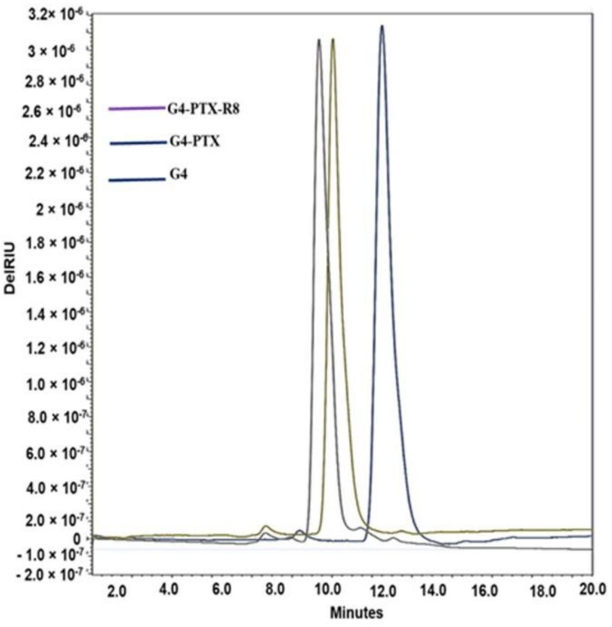

Figure S3: GPC thermogram of G4, G4-PTX and G4-PTX-R8.

The role of circadian rhythm on cellular uptake/internalization

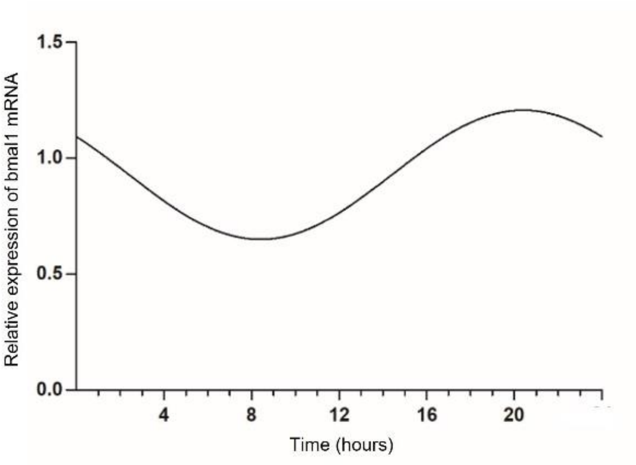

Figure S4: CircWave analysis of Bmal1 rhythmicity after HeLa cell synchronization. Relative mRNA expression levels were determined via qPCR, normalized to Cyclophilin A and cell synchronization was further confirmed by Bmal1 expression analysis by CircWave (p-value= 0.026; center of gravity= 23.38).

Table S5: Statistical analyses of Bonferroni's multiple comparisons test for PTX internalization on HeLa cells.

Bonferroni's multiple comparisons test	Significant?	Summary	Adjusted P Value
PTX			
T0 vs. T4	Yes	****	<0,0001
T0 vs. T8	Yes	****	<0,0001
T0 vs. T12	Yes	****	<0,0001
T0 vs. T16	Yes	****	<0,0001
T0 vs. T20	Yes	****	<0,0001
T4 vs. T8	Yes	****	<0,0001
T4 vs. T12	Yes	****	<0,0001
T4 vs. T16	Yes	****	<0,0001
T4 vs. T20	Yes	****	<0,0001
T8 vs. T12	Yes	****	<0,0001
T8 vs. T16	Yes	****	<0,0001
T8 vs. T20	Yes	****	<0,0001
T12 vs. T16	Yes	*	0,0107
T12 vs. T20	Yes	****	<0,0001
T16 vs. T20	No	ns	0,0505
G4-PTX-R8			
T0 vs. T4	Yes	****	<0,0001
T0 vs. T8	Yes	****	<0,0001
T0 vs. T12	Yes	****	<0,0001
T0 vs. T16	Yes	****	<0,0001
T0 vs. T20	Yes	****	<0,0001
T4 vs. T8	Yes	****	<0,0001
T4 vs. T12	Yes	****	<0,0001
T4 vs. T16	Yes	****	<0,0001
T4 vs. T20	Yes	****	<0,0001
T8 vs. T12	Yes	****	<0,0001
T8 vs. T16	Yes	****	<0,0001
T8 vs. T20	Yes	****	<0,0001
T12 vs. T16	Yes	****	<0,0001
T12 vs. T20	Yes	****	<0,0001
T16 vs. T20	Yes	****	<0,0001
PTX - G4-PTX-R8			
T0	Yes	****	<0,0001
T4	Yes	****	<0,0001
T8	Yes	****	<0,0001
T12	Yes	****	<0,0001
T16	Yes	****	<0,0001
T20	Yes	****	<0,0001

Table S6: Results of CircWave analysis of cell-associated PTX absorbance for each time-point.

		PTX	G4-PTX-R8
HeLa	CG	10	8.84
	P-value	0	2.1 E-5

CG, center of gravity.

Generation of a Bmal1 knockout cell line

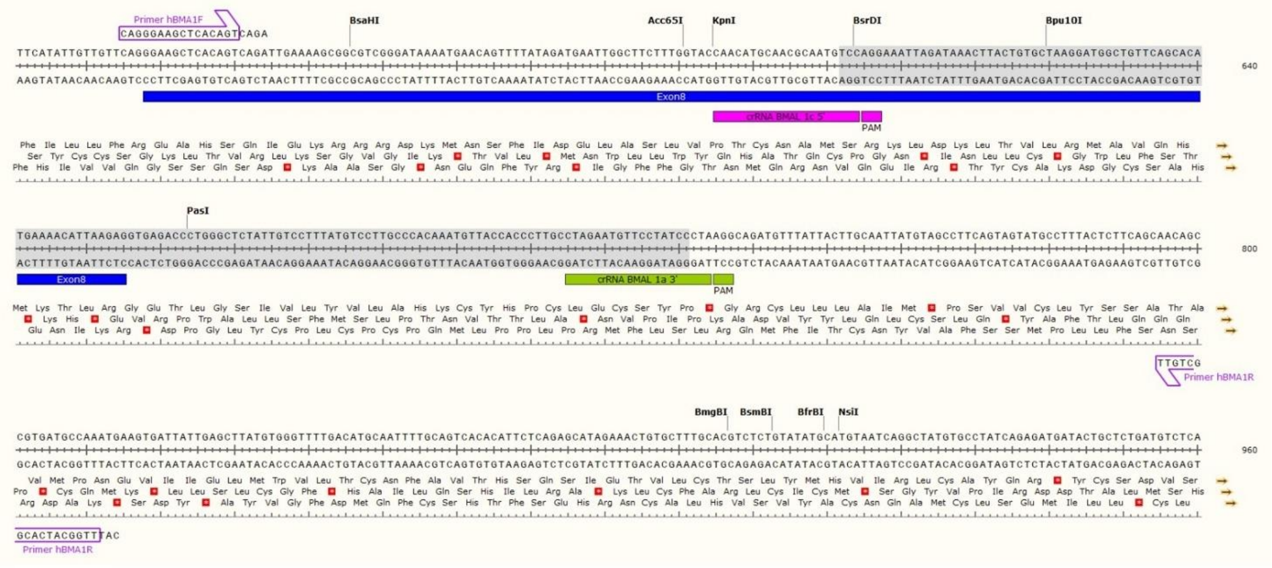

(a)

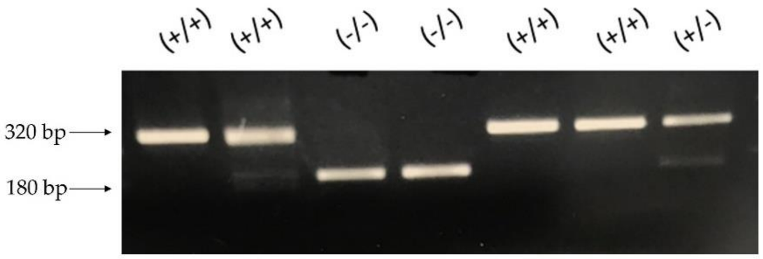

(b)

Figure S5: Genomic map of Bmal1 showing the sequence of the deleted fragment from exon 8 in grey (a) and RT-PCR products of the isolated clones (b). We isolated 2 cell lines with Bmal1 Knockout (-/-) and 2 with a "heterozygous" deletion (HET, (+/-)).

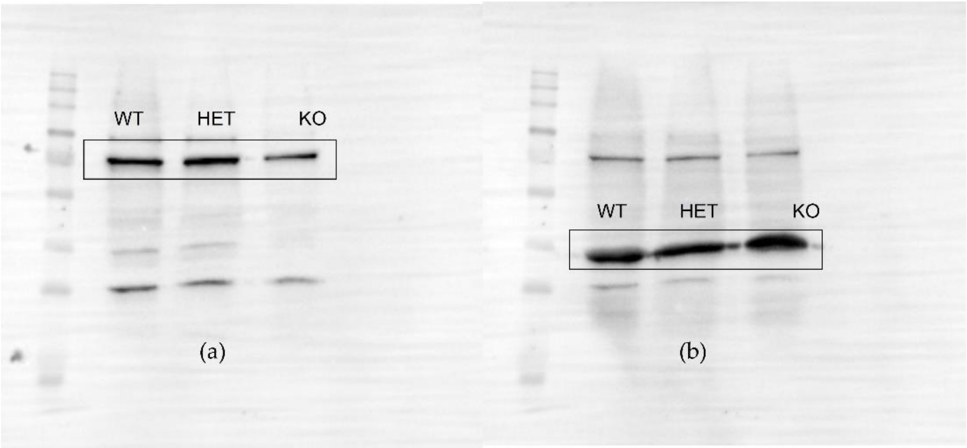

Figure S6: Western blot analysis of BMAL1 protein expression in HeLa cells. A single band of 75 kDa corresponding to BMAL1 was observed in electroporated cells. A lower level of BMAL expression was observed in HeLa knockout cells (a). Beta-actin expression of the cells was used as the loading control (b).

Bmal1 silencing effect on cellular internalization of PTX

Table S7: Statistical analyses of Bonferroni's multiple comparisons test for PTX and G4-PTX-R8 internalization on HeLa knockout cells.

Bonferroni's multiple comparisons test	Significant?	Summary	Adjusted P Value
PTX			
T0 vs. T4	Yes	****	<0,0001
T0 vs. T8	Yes	****	<0,0001
T0 vs. T12	Yes	****	<0,0001
T0 vs. T16	Yes	****	<0,0001
T0 vs. T20	Yes	****	<0,0001
T4 vs. T8	Yes	****	<0,0001
T4 vs. T12	No	ns	>0,9999
T4 vs. T16	Yes	*	0,0187
T4 vs. T20	No	ns	>0,9999
T8 vs. T12	Yes	****	<0,0001
T8 vs. T16	Yes	****	<0,0001
T8 vs. T20	Yes	****	<0,0001
T12 vs. T16	No	ns	0,7135
T12 vs. T20	No	ns	>0,9999
T16 vs. T20	Yes	**	0,0059

G4-PTX-R8			
T0 vs. T4	Yes	****	<0,0001
T0 vs. T8	Yes	****	<0,0001
T0 vs. T12	Yes	****	<0,0001
T0 vs. T16	Yes	****	<0,0001
T0 vs. T20	Yes	*	0,0390
T4 vs. T8	Yes	****	<0,0001
T4 vs. T12	Yes	****	<0,0001
T4 vs. T16	Yes	****	<0,0001
T4 vs. T20	Yes	****	<0,0001
T8 vs. T12	Yes	*	0,0271
T8 vs. T16	Yes	****	<0,0001
T8 vs. T20	Yes	****	<0,0001
T12 vs. T16	No	ns	0,1113
T12 vs. T20	Yes	****	<0,0001
T16 vs. T20	Yes	****	<0,0001
Wild-type-knockout - PTX			
T0	No	ns	>0,9999
T4	Yes	****	<0,0001
T8	Yes	****	<0,0001
T12	Yes	****	<0,0001
T16	No	ns	0,8709
T20	No	ns	>0,9999
Wild-type-knockout – G4-PTX-R8			
T0	Yes	****	<0,0001
T4	Yes	****	<0,0001
T8	Yes	****	<0,0001
T12	Yes	****	<0,0001
T16	Yes	****	<0,0001
T20	No	ns	0,0812

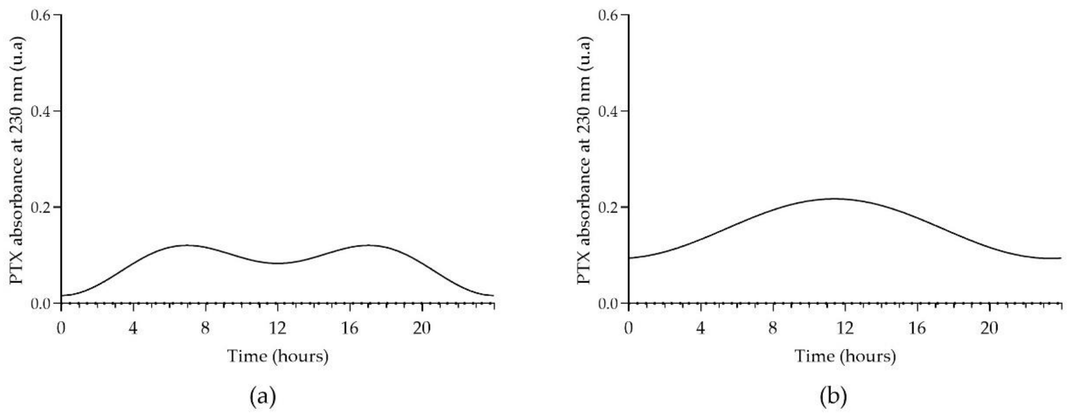

Figure S7. Circadian oscillations are statistically significant as analyzed with CircWave for PTX (a) (CG= 12; (p = 0.00079)) and G4-PTX-R8 complex (b) (CG= 11,4; (p = 1.4E-0.5)).

Table S8: Statistical analyses of Bonferroni's multiple comparisons test for caspase activity on wild-type and knockout after incubation with free PTX or G4-PTX-R8.

Bonferroni's multiple comparisons test	Significant?	Summary	Adjusted P Value
Wild-type Caspase 3			
PTX			
T0 vs. T4	No	ns	>0,9999
T0 vs. T8	Yes	****	<0,0001
T0 vs. T12	Yes	****	<0,0001
T0 vs. T16	Yes	****	<0,0001
T0 vs. T20	Yes	***	0,0003
T4 vs. T8	Yes	****	<0,0001
T4 vs. T12	Yes	****	<0,0001
T4 vs. T16	Yes	****	<0,0001
T4 vs. T20	Yes	**	0,0051
T8 vs. T12	Yes	****	<0,0001
T8 vs. T16	Yes	****	<0,0001
T8 vs. T20	Yes	****	<0,0001
T12 vs. T16	No	ns	>0,9999
T12 vs. T20	No	ns	0,1165
T16 vs. T20	Yes	**	0,0051
G4-PTX-R8			
T0 vs. T4	No	ns	0,2644
T0 vs. T8	Yes	****	<0,0001
T0 vs. T12	Yes	****	<0,0001
T0 vs. T16	No	ns	0,4490
T0 vs. T20	No	ns	0,1944
T4 vs. T8	Yes	****	<0,0001

T4 vs. T12	Yes	***	0,0003
T4 vs. T16	No	ns	>0,9999
T4 vs. T20	Yes	****	<0,0001
T8 vs. T12	Yes	****	<0,0001
T8 vs. T16	Yes	****	<0,0001
T8 vs. T20	Yes	****	<0,0001
T12 vs. T16	Yes	***	0,0002
T12 vs. T20	Yes	****	<0,0001
T16 vs. T20	Yes	***	0,0001

Wild-type Caspase 9

PTX			
T0 vs. T4	No	ns	>0,9999
T0 vs. T8	Yes	****	<0,0001
T0 vs. T12	Yes	****	<0,0001
T0 vs. T16	No	ns	>0,9999
T0 vs. T20	No	ns	>0,9999
T4 vs. T8	Yes	****	<0,0001
T4 vs. T12	Yes	****	<0,0001
T4 vs. T16	No	ns	>0,9999
T4 vs. T20	No	ns	>0,9999
T8 vs. T12	No	ns	>0,9999
T8 vs. T16	Yes	****	<0,0001
T8 vs. T20	Yes	****	<0,0001
T12 vs. T16	Yes	****	<0,0001
T12 vs. T20	Yes	****	<0,0001
T16 vs. T20	No	ns	>0,9999
G4-PTX-R8			
T0 vs. T4	Yes	****	<0,0001
T0 vs. T8	Yes	****	<0,0001
T0 vs. T12	Yes	****	<0,0001
T0 vs. T16	Yes	****	<0,0001
T0 vs. T20	Yes	*	0,0124
T4 vs. T8	Yes	****	<0,0001
T4 vs. T12	Yes	****	<0,0001
T4 vs. T16	Yes	****	<0,0001
T4 vs. T20	Yes	****	<0,0001
T8 vs. T12	Yes	****	<0,0001
T8 vs. T16	Yes	****	<0,0001
T8 vs. T20	Yes	****	<0,0001
T12 vs. T16	Yes	****	<0,0001
T12 vs. T20	Yes	****	<0,0001
T16 vs. T20	Yes	***	0,0003

knockout Caspase 3

PTX	No	ns	>0,9999
T0 vs. T4	Yes	****	<0,0001
T0 vs. T8	No	ns	0,6470
T0 vs. T12	Yes	*	0,0250
T0 vs. T16	No	ns	>0,9999
T0 vs. T20	Yes	****	<0,0001
T4 vs. T8	No	ns	0,9628
T4 vs. T12	Yes	*	0,0429
T4 vs. T16	No	ns	>0,9999
T4 vs. T20	Yes	****	<0,0001
T8 vs. T12	Yes	****	<0,0001
T8 vs. T16	Yes	****	<0,0001
T8 vs. T20	No	ns	>0,9999
T12 vs. T16	No	ns	>0,9999
T12 vs. T20	No	ns	0,0821
T16 vs. T20	No	ns	0,0821

G4-PTX-R8			
T0 vs. T4	Yes	****	<0,0001
T0 vs. T8	No	ns	>0,9999
T0 vs. T12	Yes	****	<0,0001
T0 vs. T16	Yes	****	<0,0001
T0 vs. T20	Yes	****	<0,0001
T4 vs. T8	Yes	****	<0,0001
T4 vs. T12	No	ns	0,2169
T4 vs. T16	Yes	****	<0,0001
T4 vs. T20	Yes	****	<0,0001
T8 vs. T12	Yes	****	<0,0001
T8 vs. T16	Yes	****	<0,0001
T8 vs. T20	Yes	****	<0,0001
T12 vs. T16	Yes	****	<0,0001
T12 vs. T20	Yes	****	<0,0001
T16 vs. T20	Yes	*	0,0189

knockout Caspase 9

PTX			
T0 vs. T4	No	ns	>0,9999
T0 vs. T8	Yes	****	<0,0001
T0 vs. T12	Yes	****	<0,0001
T0 vs. T16	No	ns	>0,9999
T0 vs. T20	No	ns	>0,9999
T4 vs. T8	Yes	****	<0,0001
T4 vs. T12	Yes	****	<0,0001
T4 vs. T16	No	ns	>0,9999

T4 vs. T20	No	ns	>0,9999
T8 vs. T12	No	ns	>0,9999
T8 vs. T16	Yes	***	0,0002
T8 vs. T20	Yes	***	0,0004
T12 vs. T16	Yes	****	<0,0001
T12 vs. T20	Yes	****	<0,0001
T16 vs. T20	No	ns	>0,9999
G4-PTX-R8			
T0 vs. T4	Yes	****	<0,0001
T0 vs. T8	Yes	****	<0,0001
T0 vs. T12	Yes	****	<0,0001
T0 vs. T16	Yes	****	<0,0001
T0 vs. T20	Yes	****	<0,0001
T4 vs. T8	Yes	****	<0,0001
T4 vs. T12	Yes	****	<0,0001
T4 vs. T16	Yes	****	<0,0001
T4 vs. T20	Yes	****	<0,0001
T8 vs. T12	Yes	****	<0,0001
T8 vs. T16	Yes	****	<0,0001
T8 vs. T20	Yes	****	<0,0001
T12 vs. T16	Yes	**	0,0023
T12 vs. T20	Yes	****	<0,0001
T16 vs. T20	Yes	**	0,0023
Wild-type-knockout Caspase 3			
G4-PTX-R8	Yes	**	0,0068
T0	Yes	****	<0,0001
T4	Yes	****	<0,0001
T8	Yes	****	<0,0001
T12	Yes	****	<0,0001
T16	Yes	**	0,0068
T20	Yes	****	<0,0001
Wild-type-knockout Caspase 9			
G4-PTX-R8			
	Yes	****	<0,0001
T0	Yes	****	<0,0001
T4	Yes	****	<0,0001
T8	Yes	****	<0,0001
T12	Yes	****	<0,0001
T16	Yes	****	<0,0001
T20	Yes	****	<0,0001

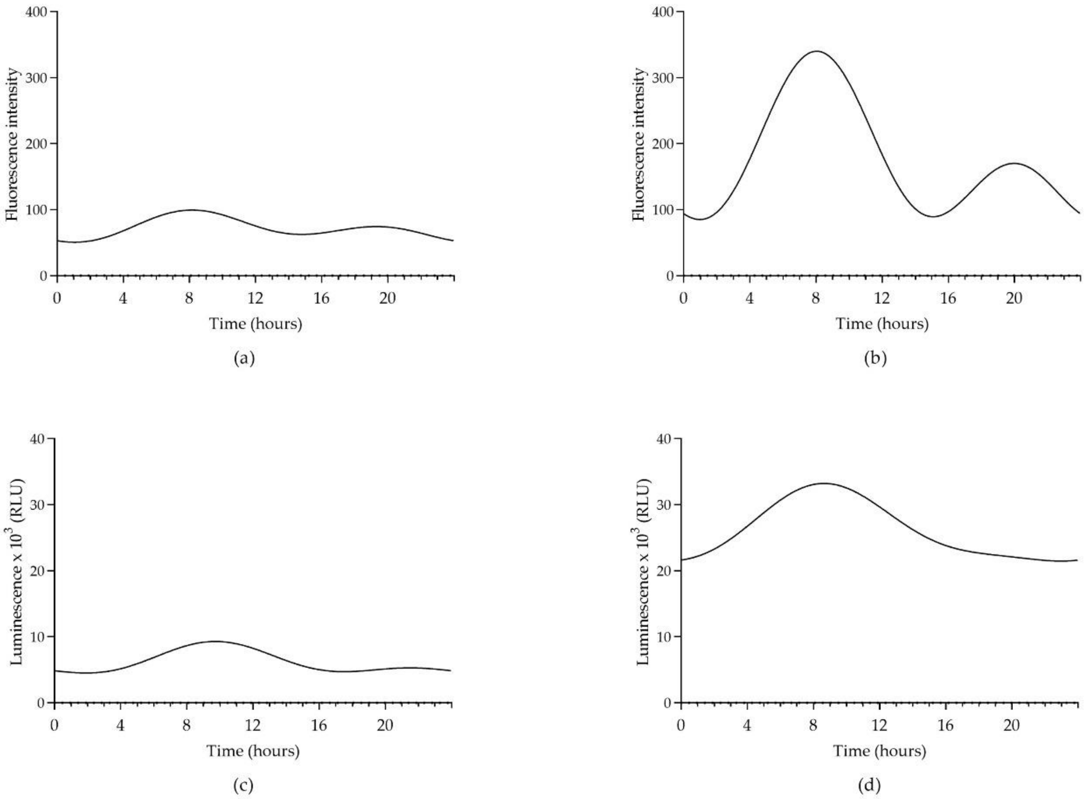

Figure S8. Circadian oscillations are statistically significant as analyzed with CircWave for caspase-3 activity after incubation with PTX (a) (CG=9.5427; p=0.001157) and G4-PTX-R8 (b) (CG= 9.8728; p=0) and for caspase-9 activity after incubation with PTX (c) (CG=9; p=0.000146) and G4-PTX-R8 (d) (CG= 8.1212; p= 0.0010).
